# Dental fluorosis and self-reported depression among young adults in the United States

**DOI:** 10.3389/fenvh.2026.1825971

**Published:** 2026-06-23

**Authors:** Angela M. Miles, Durdana Khan, Alexandra Mattia, Zhilin Wang, Ashley J. Malin

**Affiliations:** 1 College of Public Health and Health Professions, University of Florida, Gainesville, FL, United States; 2 College of Medicine, University of Florida, Gainesville, FL, United States; 3 Department of Psychology, Faculty of Health, York University, Toronto, ON, Canada

**Keywords:** dental fluorosis, depression, fluoride, mental health, NHANES, young adults

## Abstract

**Introduction:**

Chronic low-level fluoride exposure has been associated with depressive symptoms in animals and child internalizing behaviors in the United States (US); however, this study is the first to examine whether excess early life fluoride exposure is associated with depressive symptoms in US adults.

**Methods:**

Participants were 886 adults aged 18–29 years from the National Health and Nutrition Examination Survey (NHANES) 2015–2016. Dental fluorosis, a marker of excess fluoride exposure during tooth development, was assessed using the Dean’s Fluorosis Index (DFI). Self-reported depression was measured using the Patient Health Questionnaire (PHQ-9). We classified participants as having depression if they scored ≥10 and satisfied certain clinical criteria. We also classified depression symptom severity. Survey-weighted and covariate-adjusted binomial and multinomial logistic regression examined associations of dental fluorosis with presence and severity of depression, respectively.

**Results:**

Participants were 24 years-old on average. Approximately 70% of participants had dental fluorosis that was mostly very mild or mild and 5% reported depression. Participants with dental fluorosis had 1.8 times the odds of reporting current depression compared to those without fluorosis (OR = 1.78; 95% CI: 1.03–3.07; *p* = 0.04). This association was significant for very mild/mild fluorosis and depression (OR = 1.82, 95% CI: 1.06–3.11, *p* = 0.031) but not moderate/severe fluorosis and depression (*p* = 0.814). Dental fluorosis was not associated with depressive symptom severity.

**Discussion:**

Having very mild or mild dental fluorosis was associated with higher odds of young adults in the US reporting depressive symptoms; however, prospective studies are needed to further examine this association.

## 1 Introduction

Fluoride naturally occurs in rock and soil and can be released environmentally through industrial activities. It is widely used in dental products and United States (US) public water supplies for preventing dental caries (1-3). Fluoride increases resistance to dental caries by replacing hydroxyl ions in tooth hydroxyapatite, creating more crystalized and durable structures, particularly when applied topically (4, 5). However, concerns have been raised recently that children living in the US may have excess fluoride exposure, given the increasing prevalence of dental fluorosis; a marker of excess fluoride ingestion during tooth development (i.e., birth to 8 years of age) (6, 7). As such, in 2015, the US Public Health Service lowered the recommended fluoride concentration for community water fluoridation from a range of 0.7 –1.2 mg/L to 0.7 mg/L (7).

Maintaining a balance between the benefits of fluoride and its potential negative systemic health effects remains critical. Recent findings from epidemiological studies suggest that fluoride exposure, even at US-population relevant levels, may increase risk of internalizing symptoms in children and adolescents (8-10). Furthermore, the National Toxicology Program (NTP) published a systematic review and meta-analysis on neurocognitive effects of fluoride that found a consistent inverse association between fluoride exposure and child IQ (11). However, the review highlighted a lack of studies on fluoride exposure and adult cognitive and behavioral outcomes, noting that as of 2023 only two high quality studies exist (3, 12, 13).

Depression is highly prevalent and remains a leading cause of disability in the US (14). Data from the National Health and Nutrition Examination Survey (NHANES) indicates that the prevalence of depression is increasing among US adults aged 20–39 years (15, 16). Furthermore, recent research from NHANES shows that environmental toxicant exposures, including cadmium, manganese, volatile organic compound metabolites, and polycyclic aromatic hydrocarbons, are associated with depressive symptoms in adulthood (17). However, no US studies have considered fluoride. Animal studies have observed increased depressive symptoms from high levels of sodium fluoride administered in drinking water prenatally, post weaning or during adulthood (18, 19), as well as from low levels administered during gestation and lactation (20). However, to our knowledge, only one study, conducted in China, has examined the association between fluoride exposure and depression in adults, and it found no general association between creatinine-adjusted urinary fluoride levels and depression symptoms (21). The current study is the first human study to examine whether early life fluoride exposure is associated with depression in adulthood. It uniquely explores whether dental fluorosis, a marker of excess fluoride exposure during tooth development, is associated with self-reported depression in young US adults.

## 2 Materials and methods

### 2.1 Participants

This cross-sectional study included participants from the National Health and Nutrition Examination Survey (NHANES) 2015–2016 cycle, as this was the only cycle that had publicly available data on dental fluorosis in adults. Furthermore, while the 2013–2014 and 2015–2016 cycles assessed fluoride levels in household drinking water and plasma, and the 2015–2016 cycle assessed fluoride in urine, these data were only available for children and adolescents up to age 19 years. NHANES collects information during face-to-face household interviews and conducts physical examinations and administers additional self-report measures at the mobile examination center (MEC). There were 9,544 participants that completed both the interview and MEC visits. This study included participants aged 18–29 years because both dental fluorosis and self-reported depression data were available for this age range (*n* = 1,074). After excluding participants with missing covariate data (*n* = 188), the final unweighted sample included 886 participants (weighted 38,171,855) (see Figure 1 for a participant selection flow diagram). We retained 39 participants taking anti-depressant medication in the primary analysis to prevent selection bias (i.e., excluding potentially more severe cases) (22). This study was determined to be nonhuman and therefore exempt from IRB review by the University of Florida (Protocol#: NH00042926).

### 2.2 Measures

#### 2.2.1 Dental fluorosis

Dental fluorosis was assessed during a dental exam, under standardized conditions, using the Dean’s Fluorosis Index (DFI) (23). The DFI evaluates fluorosis severity across six categories, ranging from “very mild” to “severe” fluorosis based on the area of the tooth surface with visible fluorosis and the presence of pitting. Categories of dental fluorosis include normal [translucent, smooth, glossy, pale creamy white (DFI = 0)], questionable tooth [slight aberrations, a few white spots (DFI = 5)], very mild fluorosis [≤25% of tooth has small, white areas (DFI = 1.0)], mild fluorosis [>25%–<50% of the tooth has white areas (DFI = 2.0)], moderate fluorosis [≥50% of the tooth with all surfaces involved, with or without brown stains (DFI = 3.0)], and severe fluorosis [all enamel is involved and has discrete or confluent pitting (DFI = 4.0)]. The dental fluorosis value was based on the second most affected tooth. Therefore, the participant’s fluorosis status was determined by the less affected tooth if the 2 most affected teeth were not equally affected (23). We recoded questionable fluorosis to DFI = 0.5. Teeth that were missing, primary (deciduous), not fully erupted permanent teeth, or those with more than half of their visible surface area obscured by restorations, caries, or orthodontic appliances were recorded as “cannot be assessed” (DFI = 9). Additionally, teeth may exhibit non-fluoride opacities (DFI = 8), which mimic fluorosis but arise from other causes. We excluded codes 8 and 9 from the assessment (23). We created a binary indicator variable of “no fluorosis” compared to “fluorosis”. We also created a three-category variable of “no fluorosis” compared to “very mild/mild fluorosis” or “moderate/severe fluorosis”. Participants with a DFI score of ≤0.5 (questionable or no fluorosis) were categorized as not having fluorosis, while those with a DFI score of ≥1.0 were classified as having fluorosis.

Inter-examiner reliability statistics for dental fluorosis scoring ranged from moderate to near perfect (0.51–0.98) for the nine primary dental examiners based on Landis and Koch, as well as mostly adequate agreement (33 out of 36 Kappas ≥ 0.6) based on McHugh (23). Examiners also highly agreed in defining very mild or greater fluorosis. However, there was a low proportion of the total number of examinations for which a gold standard examination was conducted to derive these estimates (i.e., in which a reference examiner randomly replicated an exam). Moreover, the DFI scoring method has high examiner subjectivity, and therefore, the NCHS expressed caution about deriving estimates of prevalence and trends across survey years based on this data (23). Nevertheless, risk of dental fluorosis in NHANES 2013–2016 has been shown to correlate with higher levels of fluoride in household drinking water and plasma. In particular, prior research has observed that children and adolescents in NHANES 2015–2016 with household water fluoride levels of 0.51–0.70 as well as >0.70 mg/L had approximately double the odds of exhibiting dental fluorosis when compared to those receiving tap water with fluoride levels ≤0.30 mg/L (24, 25).

#### 2.2.2 Self-reported depression

Depression was assessed via the Patient Health Questionnaire (PHQ-9), a validated 9-item self-report measure administered by trained interviewers as part of the MEC visit using the Computer-Assisted Personal Interview (CAPI) system (26). The PHQ-9 assesses depressive symptoms over the past 2 weeks based on the mood module of the *Diagnostic and Statistical Manual of Mental Disorders, Fourth Edition* (DSM-IV) criteria. Participants are asked to rate depressive symptoms from 0 to 3 in terms of how frequently they are experienced (0 = “not at all,” 1 = “several days,” 2 = “more than half the days,” 3 = “nearly every day”). Total PHQ-9 scores range from 0 to 27 with higher scores indicating more severe depressive symptoms. The PHQ-9 has strong internal consistency, with Cronbach’s *α* exceeding 0.85 in two independent studies; validity is supported by an area under curve (AUC) of 0.95. Furthermore, the PHQ-9 has excellent sensitivity (0.88) and specificity (0.85) (26).

We applied the PHQ-9 “Treatment Selection and Monitoring” scoring approach to calculate depression scores (26, 27). This approach enables calculation of the presence of depression as well as depressive symptom severity. We classified scores of ≥10 as meeting criteria for depression among participants who reported either feeling little interest or pleasure in doing things (question 1) or feeling down, depressed or hopeless (question 2), at least more than half of the days. Additionally, they reported at least some difficulty in daily functioning due to depressive symptoms (question 10). The PHQ-9 categorizes depression severity scores as: “Minimal Symptoms” = 5–9; “Minor depression, dysthymia, or major depression, mild” = 10–14; “Major depression, moderately severe” = 15–19; and “Major depression, severe” = 20–27. We recategorized these scores as normal (i.e., no depression; score < 5); minimal symptoms/mild depression (score 5–14); and moderately severe/severe depression (score ≥ 15) to examine associations between fluorosis and depression severity.

### 2.3 Covariates

We selected covariates using a directed acyclic graph (DAG) approach and based on previous literature (28-37) (see Figure 2). Covariates associated with fluoride exposure/fluorosis and depression included age, sex, race/ethnicity, the ratio of family income to poverty, body mass index (BMI), and serum cotinine, a primary metabolite of nicotine. Race/ethnicity was self-reported and categorized as Mexican American, other Hispanic, non-Hispanic White, non-Hispanic Black, non-Hispanic Asian, and other race, including multiracial. The ratio of family income to poverty reflects socioeconomic status and was calculated as the ratio of family income to the federal poverty threshold. Scores range from 0 to 5 and higher values reflect greater financial resources. BMI was calculated as $\frac{weight\;(kg)}{(height\;(m))2}$ and included in statistical analysis as a continuous measure (38). Serum cotinine (ng/mL) was used as a biomarker for tobacco exposure.

### 2.4 Statistical analysis

We examined survey-weighted univariate statistics for dental fluorosis, depression and covariates. Continuous normally distributed variables were described with means and standard deviations (SD). Categorical variables were presented as frequencies and percentages. We conducted survey-weighted and covariate-adjusted binomial and multinomial logistic regression to examine associations between dental fluorosis and the presence or severity of depression, respectively. We conducted models that included dental fluorosis as a binary variable (fluorosis yes vs. no) or a three-category variable (very mild/mild or moderate/severe fluorosis, compared to no fluorosis). We calculated odds ratios (OR) and 95% confidence intervals (CI) for these models. For logistic regression models, we performed diagnostic tests including multicollinearity checks, goodness of fit tests, and residual analyses to ensure the validity of the model assumptions. These tests indicated no collinearity concerns, and no influential outliers based on Cook’s distance.

Primary models were adjusted for age, sex, race/ethnicity, the ratio of family income to poverty, BMI, and serum cotinine. We also conducted sensitivity analyses additionally adjusting for adiposity and alcohol use; however, alcohol use data was only available in a subsample of 637 participants. We created a binary BMI variable of obese (BMI ≥ 30) and not obese (BMI < 30) to distinguish between high and low adiposity, rather than adjusting for continuous BMI. To determine alcohol consumption patterns, participants were asked to report the number of drinks they consumed per day, with a drink defined as a 12 oz. beer, a 5 oz. glass of wine, or 1.5 oz. of liquor. We categorized alcohol consumption as “typical” = 0–2 drinks per day and “atypical” = more than 2 drinks per day because excessive alcohol consumption can contribute to depressive symptoms and negatively impact dental health, including contributing to tooth staining, which could potentially impact fluorosis scoring (39). We also conducted a sensitivity analysis excluding all participants who reported taking antidepressant medication (*n* = 39). NHANES collects information on all prescription medications taken in the past 30 days during the household interview, with interviewers verifying medication containers whenever available (40, 41). We created a binary variable (yes/no) reflecting whether participants reported using any antidepressant medication during the 30-day reference period. Lastly, we tested interactions of binary fluorosis with age, sex, race/ethnicity, income and continuous BMI to examine whether the association between dental fluorosis and depression was modified by these variables. Interaction terms were to be retained in the model if statistically significant. An alpha (*α*) of <.05 was the threshold for statistical significance. All models applied survey weights from the MEC visit. Statistical analyses were performed using R version 4.4.1 (R Core Team R, 2024) and Stata/MP version 18.5 (Stata Corp, 2025).

## 3 Results

Descriptive statistics for sociodemographic characteristics are presented in Table 1. The mean (SD) age was 23.99 (3.24) years and there was nearly an equal distribution of males (52.13%) and females (47.86%). Most participants were Non-Hispanic White (56.61%). Approximately 70% of participants had dental fluorosis, with 67% exhibiting very mild or mild cases. Approximately 5% of participants reported symptoms consistent with depression. In terms of depression severity, 5% of participants reported minimal symptoms or mild depression, while 2% reported moderate or severe depressive symptoms.

Associations between dental fluorosis and the odds of reporting depression are presented in Table 2. Participants with dental fluorosis had 1.78 times higher odds of reporting depression compared to those without dental fluorosis (OR = 1.78, 95% CI: 1.03–3.07, *p* = 0.039). When fluorosis was examined categorically, participants with very mild/mild fluorosis had higher odds of reporting depression (OR = 1.82, 95% CI: 1.06–3.11, *p* = 0.031) while those with moderate/severe fluorosis did not (OR = 0.80, 95% CI: 0.12–5.55, *p* = 0.814). Results were consistent when excluding 39 participants taking antidepressant medication (OR = 3.06, 95% CI: 1.04–8.95, *p* = 0.042 for having fluorosis compared to no fluorosis; OR = 3.09, 95% CI: 1.06–9.02, *p* = 0.040 for very mild/mild fluorosis compared to no fluorosis); however, magnitudes of association were larger, and confidence intervals were wider (Supplementary Table S1). Having dental fluorosis was not significantly associated with depressive symptom severity, although magnitudes of associations were positive (OR = 1.23, 95% CI: 0.56–2.69, *p* = 0.590 for minimal/mild depressive symptoms, and OR = 1.03, 95% CI: 0.27–3.98, *p* = 0.959 for moderate/severe depressive symptoms, when compared to participants with normal mood). Results were consistent when fluorosis was examined as a three-category variable in relation to depression severity (Table 3). None of the interactions tested were statistically significant in any of the models.

Associations between dental fluorosis and self-reported depression remained significant after adjusting for adiposity categorically, rather than BMI continuously, in sensitivity analyses (Supplementary Table S2). When adjusting for alcohol consumption in a reduced sample of 637 participants (weighted *N* = 29,801,410), associations between dental fluorosis and depression were still positive but attenuated in magnitude and no longer statistically significant (Supplementary Table S3). However, when rerunning the primary model examining the association between dental fluorosis and depression in the reduced sample of 637 participants, but without adjusting for alcohol use, the association was also attenuated and non-significant. Furthermore, the association between dental fluorosis and minimal symptoms/mild depression was similar to that in the model adjusting for alcohol use. This points to a loss of power, rather than confounding by alcohol use, attenuating the association (Supplementary Table S4). Demographic characteristics, and prevalence of fluorosis and depression were similar between the overall sample and sample with alcohol use data (Supplementary Table S5).

## 4 Discussion

This study found that adults with very mild or mild dental fluorosis had nearly double the odds of reporting symptoms consistent with current depression when compared to adults without dental fluorosis. Therefore, young adults who had excess fluoride exposure during tooth development (i.e., birth to 8 years of age) had higher odds of reporting depression during the 2 weeks prior to completing the PHQ-9. Some experimental research shows that chronic low-level early life fluoride exposure may contribute to neurochemical changes that can increase risk of future depression (20). However, prior research in NHANES 2015–2016 also observed higher plasma and water fluoride levels among adolescents (ages 12–19 years) who had dental fluorosis (24). This suggests that fluoride exposure may be at least somewhat stable between childhood and adulthood. Furthermore, this may point to a role of chronic fluoride exposure during youth in adult depression risk; however, NHANES 2015–2016 did not collect data on chronic fluoride exposure, and depression scores among youth are restricted from public access. Therefore, longitudinal studies are needed to further examine this question.

Although we examined categories of dental fluorosis in relation to adult depressive symptoms, we were limited in our ability to examine a dose-response relationship due to the low frequency of moderate and severe dental fluorosis, as well as moderate and severe depressive symptoms. Frequencies remained low even though we collapsed moderate and severe categories into one. The low frequency of these more severe presentations (i.e., 28 participants with moderate fluorosis and 3 participants with severe fluorosis) may also account for the lack of significant association between dental fluorosis and depression severity observed in this study. Specifically, this may have limited statistical power to detect associations. Furthermore, the inclusion of participants receiving depression treatment, whether psychopharmacologic or psychotherapeutic (which NHANES did not assess) could have contributed to reductions in depression severity and attenuated severity differences. Additional prospective studies including more moderate and severe cases along with treatment characterization are needed to further investigate these associations.

While no US-based studies have examined associations of fluoride exposure with depression in adults, some have found links between fluoride exposure and internalizing symptoms in youth (8, 9). For example, a US-based cross-sectional study of adolescents in Cincinnati, Ohio found that higher urinary fluoride levels among adolescents were associated with increased somatization and higher internalizing scores, although associations with a depression subscale in particular were not significant (8). Malin et al. (9) found that higher prenatal fluoride exposure was associated with more neurobehavioral problems, particularly in the realm of internalizing, among 3-year-old children in Los Angeles, California (9). Specifically, children whose mothers had higher levels of fluoride in their urine during pregnancy tended to exhibit more symptoms of emotional reactivity, anxiety, somatic complaints and autism behaviors (9). However, a recent cross-sectional study conducted among adults in endemic fluorosis areas in China, yielded inconsistent results (21). Specifically, Yu et al. (21) found no association between creatinine-adjusted urinary fluoride levels and depression symptoms reported on the Patient Health Questionnaire-2 among adults aged 18–60 years (21). However, they observed a significant interaction between urinary fluoride and income such that participants in the middle-income bracket had lower odds of depression as urinary fluoride levels increased when compared to those with low or high income (21). The difference between these findings and the current study findings may be due to a difference in fluoride exposure levels, as well as participant age range and the fluoride exposure window assessed. Specifically, the Chinese study assessed urinary fluoride among adults with high fluoride exposure, while the current study assessed chronic low-level fluoride exposure during childhood via dental fluorosis.

There are clear mechanisms by which fluoride exposure may contribute to symptoms of depression, as well as anxiety, as demonstrated in animal studies (20, 43, 44). Bartos et al. (20), found that pre- and perinatal fluoride exposure in male rats caused impairments in memory function, depressive-like behavior, and altered cholinergic and glutamate activity as well as increased oxidative stress by young adulthood (20). Similarly, Kivrak (43) found that adult mice exposed to high levels of fluoride exhibited increased anxiety-like behaviors, including higher defecation rates and reduced locomotor activities (43). Another study conducted in adult mice found that fluoride exposure led to alterations in hippocampal genes linked with anxiety and depression, resulting in abnormal behavior and depressive-like symptoms (18). Prenatal, neonatal or adult fluoride exposure has also been shown to contribute to neuroinflammation, which is implicated in the etiology of depression (45-49). Lastly, some human and animal studies point to a potential role of fluoride in disrupting thyroid gland function in children and adults, which is critical for mood regulation (50-53). However, not all studies have observed these effects (54).

Adjusting for adiposity did not change the results of this study. However, after adjusting for alcohol use, the magnitude of association between dental fluorosis and depression was attenuated, albeit still positive, and became nonsignificant. Nonetheless, alcohol use was only measured in a subsample of participants, and the NCHS has highlighted limitations with examining associations among subsamples in a single NHANES cycle (55). Therefore, given the exploratory nature of this study and methodological constraints around comprehensively adjusting for residual confounding, future prospective studies on this topic are needed.

### 4.1 Strengths and limitations

The study has several strengths, including a large, nationally representative US sample which increases generalizability of the findings. Additionally, it includes a validated measure of depression (i.e., the PHQ-9) and a standardized clinical dental examination conducted by trained examiners using the DFI to assess dental fluorosis (a graded biomarker of early life fluoride exposure). We also adjusted for a breadth of covariates associated with fluoride exposure/metabolism and depression in our models.

However, this study also has limitations. First, as previously stated, alcohol use was only assessed among a subsample of participants. The NCHS recommends combining survey cycles for examining associations in subsamples, particularly for outcomes that have low prevalence, yet this was not possible in the current study (55). Future prospective studies of fluoride exposure and depression that adjust for alcohol use are needed, given the complex bidirectional relationship between alcohol use and depression (42, 56). Second, the cross-sectional study design limits causal inference; however, including dental fluorosis as a proxy for fluoride exposure provides prospective exposure assessment, given that fluorosis is a marker of excess fluoride exposure during tooth development. Nevertheless, covariates were assessed around the time of the depression assessment rather than the exposure assessment, which limited our ability to adjust for covariates that may have influenced early life fluoride exposure. A prospective study design could address this limitation. Further, depression was self-reported rather than clinician assessed, which can introduce underreporting of symptoms (57-59); however, the PHQ-9 is highly reliable and has high sensitivity, making it less subject to outcome misclassification (60). Additionally, given that only approximately 3% of the sample had either moderate or severe dental fluorosis and only 2% reported moderate or severe depression, we could not examine whether a dose-response relationship exists. We also could not compare the association between dental fluorosis and depressive symptoms among treated vs. untreated individuals or adjust for treatment status in our models because NHANES does not assess engagement in psychotherapy (aside from whether the participant saw a mental health professional at all in the last year) (61). Lastly, since dental fluorosis provides a proxy for fluoride exposure during childhood, the childhood fluoride exposure levels of participants in this study would have reflected water fluoride levels from before the recommended community water fluoridation level was lowered in 2015 (7). Future studies are needed to examine associations of fluoride exposure and depression at current recommended drinking water fluoride levels.

## 5 Conclusion

In this nationally representative cross-sectional study, having very mild or mild dental fluorosis was associated with higher odds of young adults in the US reporting depressive symptoms. These findings support the importance of rigorously examining the association between chronic low-level early life fluoride exposure and depression. Furthermore, given the exploratory nature of this study, future prospective studies are needed to further examine this association and to determine whether a dose-response relationship exists.

## Supplementary Material

Frontiers Supplemental Material

The Supplementary Material for this article can be found online at: https://www.frontiersin.org/articles/10.3389/fenvh.2026.1825971/full#supplementary-material

## Figures and Tables

**FIGURE 1:**
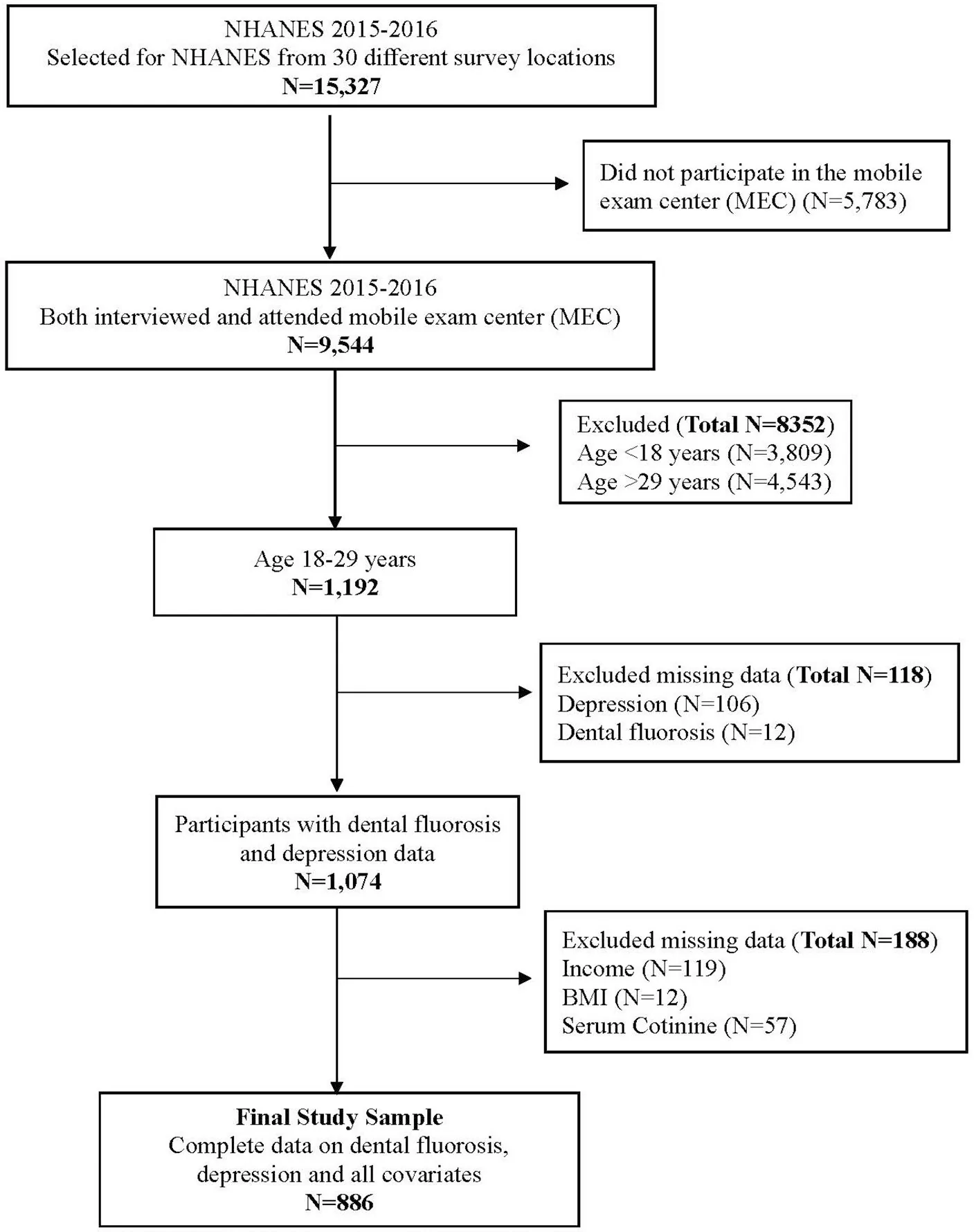
Participant selection flow diagram.

**FIGURE 2:**
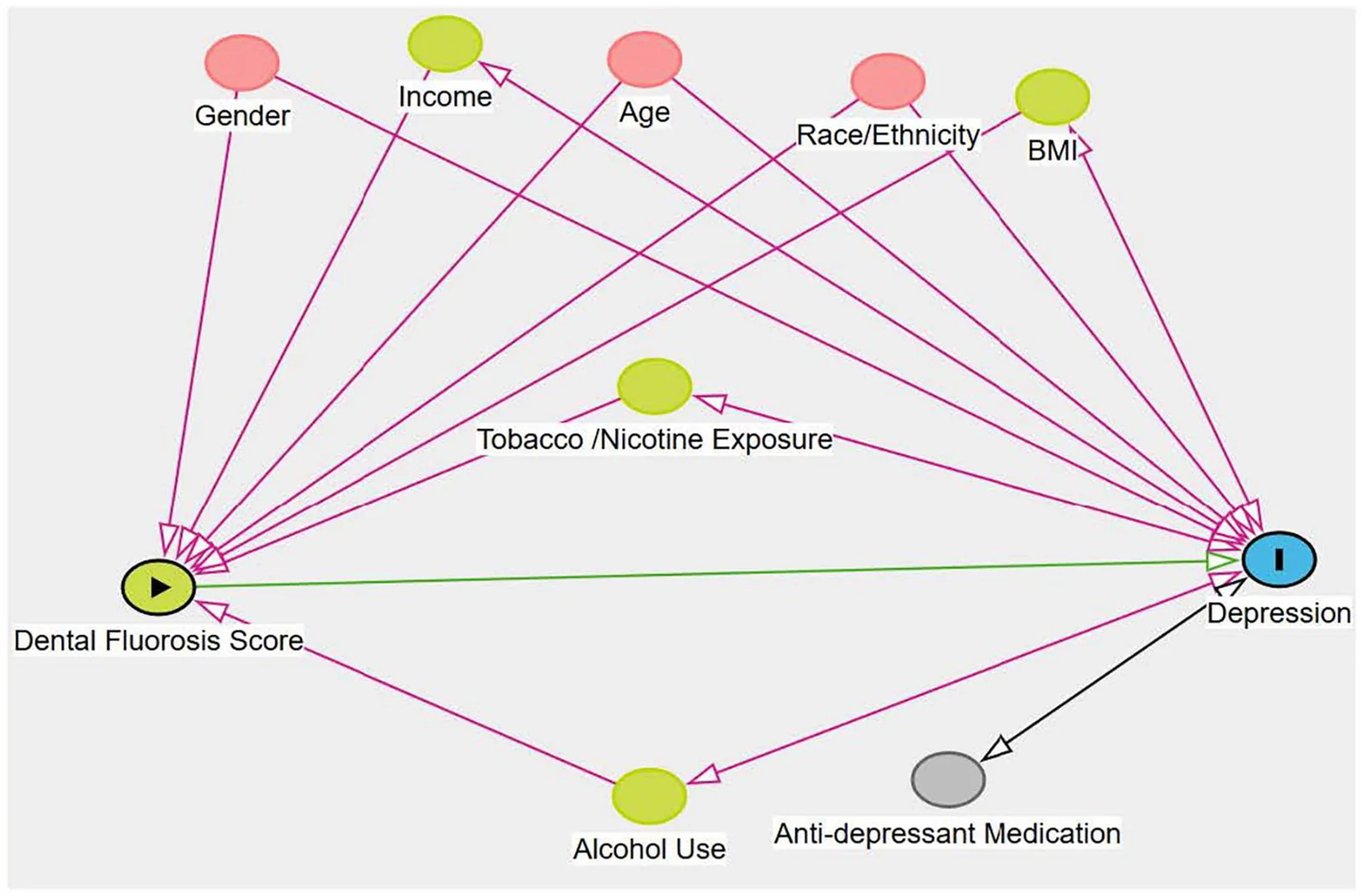
Directed acyclic graph (DAG) depicting covariate selection for logistic regression models examining associations between dental fluorosis and self-reported depression. Created using Daggity.

**TABLE 1:** Demographic characteristics of study participants aged 18–29 years.

Socio-demographic variables	Unweighted *n* = 886
	Weighted *N* = 38,171,855
Age (in years); mean (SD)	23.99 (3.24)
Sex; Freq (%)	
Male	19,901,530 (52.13)
Female	18,270,325 (47.86)
Race/Ethnicity; Freq (%)	
Mexican American	5,167,634 (13.54)
Other Hispanic	2,744,576 (7.19)
Non-Hispanic White	21,608,399 (56.61)
Non-Hispanic Black	5,000,907 (13.10)
Non-Hispanic Asian	2,209,987 (5.79)
Other race/multi-racial	1,440,349 (3.77)
Ratio of family income to poverty; mean (SD)	2.54 (1.59)
BMI (kg/m^2^); mean (SD)	27.61 (7.26)
Serum cotinine (ng/m/L); mean (SD)	42.92 (97.48)
Use of any anti-depressants in past 30 days; Freq (%)	
Yes	1,995,003 (5.23)
No	36,176,851 (94.77)
Exposure variables	
Dental fluorosis (binary); Freq (%)	
No/questionable	11,428,732 (29.94)
Yes	26,743,123 (70.06)
Dental fluorosis (three category); Freq (%)	
No/questionable	11,428,732 (29.94)
Very mild/mild	25,638,762 (67.17)
Moderate/severe	1,104,360 (2.89)
Outcome variables	
Depression (binary); Freq (%)	
No	36,417,001 (95.40)
Yes	1,754,853 (4.60)
Depression severity (categorical); Freq (%)	
Normal/no depression	35,605,763 (93.28)
Minimal symptoms/mild depression	1,753,964 (4.59)
Moderate/severe depression	812,126 (2.13)

BMI, body mass index; SD, standard deviation; Freq, frequencies. All estimates were calculated using NHANES survey MEC weights.

**TABLE 2:** Associations between dental fluorosis and the presence of self-reported depression among adults aged 18–29 years.

Exposure variable	Self-reported depression	
Dental fluorosis (binary)	Odds ratio; OR (95% CI)	*p*-value
No	1.00	
Yes	1.78 (1.03–3.07)	0.039*
Dental fluorosis (categorical)		
No/questionable	1.00	0.072^a^
Very mild/mild	1.82 (1.06–3.11)	0.031*
Moderate/severe	0.80 (0.12–5.55)	0.814

The weighted sample size is *N* = 38,171,855; the unweighted sample size is *n* = 886. For dental fluorosis severity categories, *n* = 490 participants had very mild, *n* = 94 participants had mild, *n* = 28 had moderate, and *n* = 3 had severe fluorosis, while 271 participants had questionable or no fluorosis. Logistic regression models were conducted to examine associations between dental fluorosis (predictor) and self-reported depression (outcome). Models are adjusted for age, sex, race/ethnicity, the ratio of family income to poverty, BMI and serum cotinine. The estimates, 95% CIs and *p*-values were calculated using NHANES survey MEC weights.

a The overall *p*-value for a Type 3 Analysis of Effects with 2 degrees of freedom (adjusted Wald test).

* Statistically significant at *p* < 0.05.

**TABLE 3:** Associations between dental fluorosis and self-reported depression severity among adults aged 18–29 years.

Exposure variable	Self-reported depression severity			
	Minimal symptoms/mild depression		Moderate/severe depression	
Dental fluorosis (binary)	Odds ratio OR (95% CI)	*p*-value	Odds ratio OR (95% CI)	*p*-value
No	1.00			
Yes	1.23 (0.56–2.69)	0.590	1.03 (0.27–3.98)	0.959
Dental fluorosis (categorical)				
No/questionable	1.00		1.00	0.860^a^
Very mild/mild	1.23 (0.55–2.74)	0.597	1.06 (0.28–4.05)	0.931
Moderate/severe	1.19 (0.19–7.66)	0.842	0.41 (0.04–4.16)	0.432

The weighted sample size is *N* = 38,171,855; the unweighted sample size is *n* = 886. For dental fluorosis severity categories, *n* = 490 participants had very mild, *n* = 94 participants had mild, *n* = 28 had moderate, and *n* = 3 had severe fluorosis, while 271 participants had questionable or no fluorosis. Logistic regression models were conducted to examine associations between dental fluorosis (predictor) and self-reported depression (outcome). Models are adjusted for age, sex, race/ethnicity, the ratio of family income to poverty, BMI and serum cotinine. The estimates, 95% CIs and *p*-values were calculated using NHANES survey MEC weights.

a The overall *p*-value for a Type 3 Analysis of Effects with 2 degrees of freedom (adjusted Wald test).

## Data Availability

Publicly available datasets were analyzed in this study. This data can be found here: https://www.cdc.gov/nchs/nhanes/.
