# Supplementary material for: Dental fluorosis and self-reported depression among young adults in the United States
Source: Front Environ Health. Author manuscript; Available in PMC 2026 Nov 13. (PMC13756494; doi:10.3389/fenvh.2026.1825971)
Supplement: Supplementary file 1 [file NIHMS2221352-supplement-Frontiers_Supplemental_Material.docx]

Supplementary Material

# 1 Supplementary Figures and Tables

## 1.1 Supplementary Tables

| Table S1: Associations between dental fluorosis and self-reported depression among adults aged 18-29 years, excluding participants (n=39) using antidepressant medication | | |
| --- | --- | --- |
|  | **Presence of self-reported depression** | |
| Dental fluorosis (binary) | Odds Ratio; OR (95% CI) | *p*-value |
| No | 1.00 |  |
| Yes | 3.06 (1.04-8.95) | 0.042* |
| Dental fluorosis (categorical) |  |  |
| None/questionable | 1.00 | 0.072^a^ |
| Very mild/mild | 3.09 (1.06-9.02) | 0.040* |
| Moderate/severe | 1.93 (0.20-18.89) | 0.547 |
| The weighted sample size is *N* = 36,176,851; the unweighted sample size is *n* = 847  Logistic regression models were conducted to examine associations between dental fluorosis (predictor) and self-reported depression (outcome). Models are adjusted for age, sex, race/ethnicity, the ratio of family income to poverty, BMI and serum cotinine. The estimates, 95% CIs and *p*-values were calculated using NHANES survey MEC weights  ^a^ The overall *p*-value for a Type 3 Analysis of Effects with 2 degrees of freedom (adjusted Wald test)  *Statistically significant at *p* < 0.05 | | |

| Table S2. Associations between dental fluorosis and self-reported depression adjusting for adiposity among adults aged 18-29 years | | | | | | |
| --- | --- | --- | --- | --- | --- | --- |
|  | **Self-reported Depression severity** | | | | **Self-reported Depression (Binary)** | |
|  | **Minimal Symptoms/mild depression^a^** | | **Moderate/severe depression^a^** | |  |  |
| Dental fluorosis (binary) | Odds Ratio  OR (95% CI) | *p*-value | Odds Ratio  OR (95% CI) | *p*-value | Odds Ratio  OR (95% CI) | *p*-value |
| No | 1.00 |  | 1.00 |  | 1.00 |  |
| Yes | 1.22 (0.56-2.68) | 0.596 | 1.03 (0.26-3.98) | 0.968 | 1.75 (1.01-3.05) | 0.048* |
| The weighted sample size is *N* = 38,171,855; the unweighted sample size is *n* = 886,  Logistic regression models were conducted to examine associations between dental fluorosis (predictor) and self-reported depression (outcome). These models are adjusted for age, sex, race/ethnicity, the ratio of family income to poverty, serum cotinine and adiposity. The estimates, 95% CIs and *p*-values were calculated using NHANES survey MEC weights  ^a^ The overall *p*-value for a Type 3 Analysis of Effects with 2 degrees of freedom (adjusted Wald test) is *p =* 0.843  *Statistically significant at *p* < 0.05 | | | | | | |

| Table S3. Associations between dental fluorosis and self-reported depression adjusting for alcohol use among adults aged 18-29 years | | | | | | |
| --- | --- | --- | --- | --- | --- | --- |
|  | **Self-reported Depression severity** | | | | **Self-reported Depression (Binary)** | |
|  | **Minimal Symptoms/mild depression^a^** | | **Moderate/severe depression^a^** | |  |  |
| Dental fluorosis (binary) | Odds Ratio  OR (95% CI) | *p*-value | Odds Ratio  OR (95% CI) | *p*-value | Odds Ratio  OR (95% CI) | *p*-value |
| No | 1.00 |  | 1.00 |  | 1.00 |  |
| Yes | 1.44 (0.49-4.25) | 0.481 | 0.63 (0.16-2.49) | 0.487 | 1.11 (0.57-2.17) | 0.739 |
| The weighted sample size is *N* = 29,801,410; the unweighted sample size is *n* = 637  Logistic regression models were conducted to examine associations between dental fluorosis (predictor) and self-reported depression (outcome). These models are adjusted for age, sex, race/ethnicity, the ratio of family income to poverty, BMI, serum cotinine and alcohol use  ^a^ The overall *p*-value for a Type 3 Analysis of Effects with 2 degrees of freedom (adjusted Wald test) is *p* = 0.727  The estimates, 95% CIs and *p*-values were calculated using NHANES survey MEC weights | | | | | | |

| Table S4. Associations between dental fluorosis and self-reported depression among the subsample of adults aged 18-29 years who have alcohol use data (without adjusting for alcohol use) | | | | | | |
| --- | --- | --- | --- | --- | --- | --- |
|  | **Self-reported Depression severity** | | | | **Self-reported Depression (Binary)** | |
|  | **Minimal Symptoms/mild depression^a^** | | **Moderate/severe depression^a^** | |  |  |
| Dental fluorosis (binary) | Odds Ratio  OR (95% CI) | *p*-value | Odds Ratio  OR (95% CI) | *p*-value | Odds Ratio  OR (95% CI) | *p*-value |
| No | 1.00 |  | 1.00 |  | 1.00 |  |
| Yes | 1.51 (0.55-4.10) | 0.395 | 0.88 (0.21-3.71) | 0.855 | 1.45 (0.76-2.74) | 0.24 |
| The weighted sample size is N = 29,801,410; the unweighted sample size is n = 637  Logistic regression models were conducted to examine associations between dental fluorosis (predictor) and self-reported depression (outcome). These models are adjusted for age, sex, race/ethnicity, the ratio of family income to poverty, BMI, and serum cotinine  ^a^ The overall *p*-value for a Type 3 Analysis of Effects with 2 degrees of freedom (adjusted Wald test) is *p* = 0.688  The estimates, 95% CIs and *p*-values were calculated using NHANES survey MEC weights | | | | | | |

| **Table S5. Demographic, exposure and outcome characteristics of the study sample and the subgroup with alcohol use data** | | |
| --- | --- | --- |
|  | **Overall Sample**  **Aged 18-29 years**  Unweighted n=886  Weighted N= 38,171,855 | **Alcohol Data Subgroup**  **Aged 18-29 years**  Unweighted n=637  Weighted N= 29,801,410 |
| **Socio-demographic variables** |  |  |
| **Age (in years)**; Mean (SD) | 23.99 (3.24) | 24.29 (3.01) |
| **Sex;** Freq (%) |  |  |
| Male | 19,901,530 (52.13) | 16,163,300 (54.24) |
| Female | 18,270,325 (47.86) | 13,638,110 (45.76) |
| **Race/Ethnicity**; Freq (%) |  |  |
| Mexican American | 5,167,634 (13.54) | 3,680,907 (12.35) |
| Other Hispanic | 2,744,576 (7.19) | 1,933,302 (6.48) |
| Non-Hispanic White | 21,608,399 (56.61) | 17,972,327 (60.31) |
| Non-Hispanic Black | 5,000,907 (13.10) | 3,613,384 (12.12) |
| Non-Hispanic Asian | 2,209,987 (5.79) | 1,497,926 (5.06) |
| Other race/multi-racial | 1,440,349 (3.77) | 1,103,563 (3.70) |
| **Ratio of family income to poverty**; Mean (SD) | 2.54 (1.59) | 2.62 (1.58) |
| **BMI (kg/m^2^)**; Mean (SD) | 27.61 (7.26) | 27.45 (7.12) |
| **Serum Cotinine (ng/m/L)**; Mean (SD) | 42.92 (97.48) | 49.14 (103.70) |
| **Exposure Variable** |  |  |
| **Dental Fluorosis (binary);** Freq (%) |  |  |
| No | 11,428,732 (29.94) | 9,042,580 (30.34) |
| Yes | 26,743,123 (70.06) | 20,758,829 (69.65) |
| **Outcome variables** |  |  |
| **Depression (binary);** Freq (%) |  |  |
| No | 36,417,001 (95.40) | 28,363,493 (95.17) |
| Yes | 1,754,853 (4.60) | 1,437,917 (4.82) |
| **Depression (categorical);** Freq (%) |  |  |
| Normal/no depression | 35,605,763 (93.28) | 27,718,633 (93.01) |
| Minimal symptoms/mild depression | 1,753,964 (4.59) | 1,363,047 (4.57) |
| Moderate/severe depression | 812,126 (2.13) | 719,729 (2.41) |
| BMI, Body Mass Index; SD, Standard Deviation; Freq, Frequencies  All the estimates, mean, SD, frequencies (%) were calculated using NHANES survey MEC weights | | |
